# Surgery-Induced Hippocampal Angiotensin II Elevation Causes Blood-Brain Barrier Disruption via MMP/TIMP in Aged Rats

**DOI:** 10.3389/fncel.2016.00105

**Published:** 2016-04-26

**Authors:** Zhengqian Li, Na Mo, Lunxu Li, Yiyun Cao, Wenming Wang, Yaoxian Liang, Hui Deng, Rui Xing, Lin Yang, Cheng Ni, Dehua Chui, Xiangyang Guo

**Affiliations:** ^1^Department of Anesthesiology, Peking University Third Hospital (PUTH)Beijing, China; ^2^Cancer Hospital and Institute, Chinese Academy of Medical Sciences and Peking Union Medical College, Department of Pathology, Beijing Obstetrics and Gynecology Hospital, Capital Medical UniversityBeijing, China; ^3^Department of Hematology, Peking University Third Hospital (PUTH)Beijing, China; ^4^Department of Nephrology, Peking University People’s HospitalBeijing, China; ^5^Department of Nephrology, Peking University Third Hospital (PUTH)Beijing, China; ^6^Department of Rheumatology and Immunology, Peking University Third Hospital (PUTH)Beijing, China; ^7^Key Laboratory for Neuroscience, Department of Neurobiology, Neuroscience Research Institute, Ministry of Education and Ministry of Public Health, Peking University Health Science CenterBeijing, China

**Keywords:** blood-brain barrier, matrix metalloproteinases, tissue inhibitor of matrix metalloproteinases, brain angiotensin II, postoperative cognitive dysfunction

## Abstract

Reversible blood-brain barrier (BBB) disruption has been uniformly reported in several animal models of postoperative cognitive dysfunction (POCD). Nevertheless, the precise mechanism underlying this occurrence remains unclear. Using an aged rat model of POCD, we investigated the dynamic changes in expression of molecules involved in BBB disintegration, matrix metalloproteinase-2 (MMP-2) and -9 (MMP-9), as well as three of their endogenous tissue inhibitors of MMP (TIMP-1, -2, -3), and tried to establish the correlation between MMP/TIMP balance and surgery-induced hippocampal BBB disruption. We validated the increased hippocampal expression of angiotensin II (Ang II) and Ang II receptor type 1 (AT1) after surgery. We also found MMP/TIMP imbalance as early as 6 h after surgery, together with increased BBB permeability and decreased expression of Occludin and zonula occludens-1 (ZO-1), as well as increased basal lamina protein laminin at 24 h postsurgery. The AT1 antagonist candesartan restored MMP/TIMP equilibrium and modulated expression of Occludin and laminin, but not ZO-1, thereby improving BBB permeability. These events were accompanied by suppression of the surgery-induced canonical nuclear factor-κB (NF-κB) activation cascade. Nevertheless, AT1 antagonism did not affect nuclear receptor peroxisome proliferator-activated receptor-γ (PPARγ) expression. Collectively, these findings suggest that surgery-induced Ang II release impairs BBB integrity by activating NF-κB signaling and disrupting downstream MMP/TIMP balance via AT1 receptor.

## Introduction

An aging population and new medical developments make surgery increasingly frequent in elderly patients. However, postoperative cognitive dysfunction (POCD) at advanced ages has emerged as a major health concern (Fodale et al., 2010). POCD is associated with premature departure from the workforce, increased disability, and early mortality (Steinmetz et al., 2009). The development of POCD is considered a multifactorial process, with blood-brain barrier (BBB) dysfunction and neuroinflammation cited as potential mechanisms (Terrando et al., 2011; He et al., 2012; Cao et al., 2015; Hu et al., 2014). Specifically, surgery/anesthesia disrupts BBB integrity, resulting in increased permeability, which facilitates migration of macrophages into the cerebral parenchyma and neuroinflammatory cascade in the hippocampus (Terrando et al., 2011; Cao et al., 2015), a structure critical for proper neurocognitive function. Nevertheless, the exact mechanisms underlying surgery/anesthesia-induced reversible BBB disruption remain poorly understood.

The BBB is composed of a basement membrane, interendothelial tight junctions (TJs), astrocytic end-feet, and perivascular pericytes (Pardridge, 1983). The main mediators of BBB disruption are the matrix metalloproteinases (MMPs), which are zinc-dependent endopeptidases directed against extracellular matrix proteins (Sternlicht and Werb, 2001) and TJs (Lohmann et al., 2004). Under physiological conditions, the endogenous tissue inhibitors of MMPs (TIMPs) are special inhibitors of MMPs (Yong et al., 2001), forming MMP-TIMP complexes at a ratio of 1:1, thus acting as a transcriptional regulation mechanism (Brew et al., 2000). The balance between MMPs and TIMPs plays an important role in the regulation of the normal metabolism of extracellular matrix proteins. TIMP-1 is highly inhibitory for MMP-9 (Goldberg et al., 1992), and TIMP-2 mainly inhibits MMP-2 activity and also inhibits activity of other MMP family members (Nagase, 1998). Altered MMP/TIMP balance may lead to BBB breakdown, propagation of an inflammatory response, and deposition of Alzheimer’s disease (AD)-related peptide β-amyloid (Yong et al., 2001; Mroczko et al., 2013). MMP/TIMP imbalance is therefore thought to be a key feature of the pathology of many inflammatory brain disorders, such as AD (Mroczko et al., 2013) and cerebral ischemia reperfusion injury (Wu et al., 2015).

We recently have reported that surgery enhances hippocampal angiotensin II (Ang II) content and induces BBB disruption in an animal model of POCD (Li et al., 2014). However, the downstream consequences of the surgery-induced elevation in Ang II expression, as well as the upstream mechanisms of surgery-induced BBB disruption, in the aged brain remain largely undetermined. To obtain a better understanding of BBB disruption associated with POCD, it is crucial to investigate the *in vivo* role of surgical trauma on MMP and TIMP expressions. Therefore, using an established POCD model, we dynamically examined hippocampal expression of MMP and TIMP post-surgery. We also addressed whether disturbance of the well-balanced equilibrium of MMPs and TIMPs could serve as a bridge mechanism between surgery-induced Ang II expression and postoperative BBB disruption.

## Materials and Methods

### Animals and Ethics

All experimental procedures were approved by the Peking University Biomedical Ethics Committee Experimental Animal Ethics Branch (Certification number: LA201413), and followed national guidelines (Guidelines on Administration of Laboratory Animals in China and Guidelines on the Humane Treatment of Laboratory Animals in China). Twenty-month-old male Sprague–Dawley rats (Dongchuang Laboratory Animal Center, Changsha, Hunan, China) were used. They were housed in a light-, temperature-, and humidity-controlled environment with standard laboratory chow and water *ad libitum*. Before experimental manipulations, animals were allowed at least 1 week to acclimate to the environment. All efforts were made to minimize animal number and suffering.

### Experimental Procedures

#### Experiment A

To study the effects of peripheral surgery on central Ang II levels and the normal balance between MMPs and TIMPs in the aged hippocampus, rats were randomly assigned to surgery (*n* = 30) or sham (*n* = 6) groups, and underwent laparotomy surgery under isoflurane anesthesia or received anesthesia without surgery, respectively. Animals in the sham group received no treatment in their cages. Ang II levels and MMP and TIMP gene expression were dynamically determined at 3, 6, 12, 24, and 72 h after surgery using radioimmunoassay and real-time reverse transcription PCR (qRT-PCR), respectively (*n* = 6 per time point).

#### Experiment B

In order to verify central Ang II/Ang II receptor type 1 (AT1) activity following surgery, the hippocampal samples harvested from the animals in sham group (*n* = 6) and those sacrificed at 24 h post-surgery (*n* = 6) in experiment A were used. AT1 transcriptional activity (AT1A and AT1B subtypes) was analyzed by qRT-PCR, while protein expression was assessed by western blot.

#### Experiment C

To explore the downstream pathways of Ang II/AT1 signaling, another cohort of rats were randomly assigned to sham, surgery, and CAND (candesartan) + surgery groups (*n* = 6 each). Rats in the CAND + surgery group were intraperitoneally administered with candesartan at a non-hypotensive dose of 0.1 mg/kg daily for 14 consecutive days pre-treatment. Rats in the other two groups received an identical volume of vehicle solution. Moreover, it’s important to note that candesartan, at this dosage, does not interfere with hippocampus-dependent memory function and BBB permeability in aged rats (Li et al., 2014). So the CAND alone group was not setted in this part of the experiment. Following the pretreatment phase, the animals in surgery and CAND + surgery groups received laparotomy under isoflurane anesthesia while the rats in sham group received no treatment. At 6 h post-surgery, six rats in each group were randomly selected and sacrificed by deep anesthesia, and the balance between MMPs and TIMPs at the protein level was determined. Additionally, the involvement of nuclear factor-κB (NF-κB) signaling and nuclear receptor peroxisome proliferator-activated receptor-γ (PPARγ) was also investigated.

#### Experiment D

To further determine the molecular mechanims underlying the surgery-induced BBB disruption and the therapeutic benefit of AT1 blockade, another 30 aged rats were used and randomly assigned to sham, surgery, and CAND (candesartan) + surgery groups (*n* = 10 each). The CAND pretreatment and surgery intervention were the same as Experiment C. At 24 h post-surgery, four rats in each group were sacrificed for the examation of BBB ultrastructural changes by using a transmission electronmicroscope (TEM). The remaining six animals in each group were sacrificed for the analysis of BBB permeability and protein expression of BBB components.

### Anesthesia and Surgery

Animals were endotracheally intubated and mechanically ventilated with 1–2% isoflurane in 100% oxygen as we have previously described (Li et al., 2014). We chose this anesthesia because this anesthetic protocol is clinically relevant. And many more recent animal studies and clinical observations have supplied evidence that anesthetics, particularly inhalational anesthetics (e.g., isoflurane, sevoflurane, desflurane), may play a role in cognitive decline (Vlisides and Xie, 2012; Steinmetz and Rasmussen, 2016). Isoflurane, the most-commonly used inhalational anesthetic agent globally, has been shown to induce POCD via the cytokine-dependent neuroinflammatory mechanisms (Li et al., 2013).

Laparotomy was aseptically performed using a previously described method developed as a model of POCD in aged rats (Barrientos et al., 2012). Briefly, a 3-cm vertical incision was made ×0.5 cm below the lower right rib. The surgeon inserted the index finger up to the second knuckle into the opening and vigorously manipulated the viscera and musculature. Next, approximately 10 cm of the intestine were exteriorized and vigorously rubbed between the surgeon’s thumb and index finger for 30 s. The intestines were then placed back into the peritoneal cavity. The skin was sutured with surgical staples. The surgery duration was 20–25 min.

### Radioimmunoassay

In experiment A, rats were deeply anesthetized with sodium pentobarbital (100 mg/kg) and submitted to transcardiac perfusion with 0.9% saline. Hippocampal samples were obtained immediately at 3, 6, 12, 24, and 72 h after surgery (*n* = 6 each). Ang II content was measured by radioimmunoassay using commercially available kits (North Institute of Biological Technology, Beijing, China), following the manufacturer’s instructions. Protein concentrations of hippocampal samples were determined using a BCA protein assay kit (Applygen Technologies Inc., Beijing, China). Hippocampal Ang II levels were expressed as ng/g total brain protein.

### Real-Time qPCR

In experiment A, ipsilateral hippocampi collected from sham rats and rats that received laparotomy at 3, 6, 12, 24, and 72 h after surgery (*n* = 6 each). Transcript levels of MMPs (MMP-2, MMP-9) and TIMPs (TIMP-1, TIMP-2, TIMP-3) were analyzed by qRT-PCR. In experiment B, hippocampal samples were obtained as mentioned above, and the mRNA levels of AT1 subtypes, AT_1A_ and AT_1B_, were also analyzed (*n* = 6 each). Briefly, total RNA was extracted using Eastep Universal RNA Extraction Kit (Promega, Madison, WI, USA) and the concentration was assessed using a Nanodrop spectrophotometer (Thermo Scientific, Waltham, MA, USA). Total RNA (2 μg) was reverse-transcribed using the GoScript Reverse Transcription System (Promega). The cDNA solution (2 μL) was subjected to qPCR in a Bio-Rad iCycler iQ system using the GoTaq^®^ qPCR Master Mix (Promega). Quantitative PCR consisted of 40 cycles, 15 s at 95°C and 60 s at 60°C each. Primer sequences are presented in Table 1. Data were analyzed by the 2^−ΔΔ CT^ method and expressed as fold change from the controls.

**Table 1:** **Sequences of primers used for RT-qPCR analysis**.

Gene	Forward primer (5′ – 3′)	Reverse primer (5′ – 3′)
*AT_1A_*	CTCAAGCCTGTCTACGAAAATGAG	TAGATCCTGAGGCAGGGTGAAT
*AT_1B_*	CTTTCCTACCGCCCTTCAGATA	TGAGTGCTTTCTCTGCTTCAAC
*MMP-2*	TGGTGTGGCACCACCGAGGA	CCTTGCCATCGCTTCGGCCA
*MMP-9*	AGCCGGGAACGTATCTGGA	TGGAAACTCACACGCCAGAAG
*TIMP-1*	CGAGACCACCTTATACCAGCGTTA	TGATGTGCAAATTTCCGTTCC
*TIMP-2*	GCTGGACGTTGGAGGAAAGA	TGATGCTAAGCGTGTCCCAG
*TIMP-3*	GTGGGAAAGAAGCTGGTGAAG	GCACATGGGGCATCTTACTG
*β-actin*	AGAGCTATGAGCTGCCTGAC	AATTGAATGTAGTTTCATGGATG

### Western Blotting

In experiment B, ipsilateral hippocampi were abtained for the determination of AT1 expression (*n* = 6). In experiment C, ipsilateral hippocampi collected from six rats in each group at 6 h after surgery were used to determine the following proteins: MMPs (MMP-2, MMP-9), TIMPs (TIMP-1, TIMP-2, TIMP-3), and components of NF-κB signaling (p-IKKα/β, p-IκBα, and IκBα). In experiment D, another six rats were sacrificed at 24 h after surgery, and hippocampi were obtained for the analysis of BBB-specific proteins (including basal lamina protein laminin, TJ proteins occludin and zonula occludens-1 (ZO-1), and the pericyte marker PDGFRβ). Western blot analysis was performed as we previously described (Li et al., 2013). In brief, hippocampus tissues were homogenized in cold Radio-Immunoprecipitation Assay (RIPA) buffer (Applygen Technologies Inc, Beijing, China), and the quantity of protein in the supernatants was determined using a BCA protein assay kit (Applygen Technologies Inc). Protein samples (60 μg protein/lane) were separated by 8 or 10% SDS-PAGE. After transfer to membranes, the proteins were respectively probed with antibodies against AT1, MMP-2, MMP-9, TIMP-1, TIMP-2, TIMP-3, occludin, ZO-1, and PDGFRβ (1:200; Santa Cruz Biotechnology), laminin (Sigma-Aldrich, St. Louis, MO, USA), p-IKKα/β, p-IκBα, IκBα, and β-actin (1:1000; CST, Danvers, MA, USA). For detection of heavy and light chain (LC) IgG in the hippocampal tissue, blots were incubated with anti-IgG antibody (1:1000; Biosynthesis, Beijing, China). Fluorescently labeled secondary antibodies (1:10,000; LI-COR Biosciences, Lincoln, NE, USA) were used to detect primary antibody binding. Band intensities were quantified by infrared scanning densitometry (Odyssey Imaging Systems; LI-COR Biosciences). The results for rats under the different experimental conditions were normalized according to mean values of the corresponding control animals.

### Enzyme-Linked Immunosorbent Assay (ELISA)

Six rats in each group were sacrificed at 24 h post-surgery in experiment D. Arterial blood samples were immediately obtained from the abdominal aorta. Blood samples were centrifuged at 3000× g at 4°C for 10 min, and the supernatant was removed and stored at −80°C until further processing. S100β blood concentrations were determined using a commercially available quantitative ELISA kit (Kamiya Biomedical Co., Seattle, WA, USA), according to the manufacturer’s recommendations. Each experimental condition was tested in three different wells and measured in duplicate. The optical density was measured with a microplate reader (Varioskan Flash 3001, Thermo, Finland) at a 450-nm wavelength. The limit of detection for this assay was less than 13.5 pg/mL.

### Transmission Electron Microscopy (TEM)

As we described previously with minor modifications (Cao et al., 2015), hippocampi were fixed in 2.5% glutaraldehyde for 1 h, treated with 1% osmium tetroxide. Sample were then dehydrated and embedded in epoxy resin. Slice were then cut into ultrathin sections with an ultramicrotome, and contrasted with uranyl acetate and lead citrate. Specimens were observed under a electron microscopy (JEM-1400, Electron Co., Japan). Ultrastructure changes in the basal laminas, TJs, mitochondria, and the endoplasmic reticulum surrounding the capillaries were assessed by an independent observer blinded to the study.

### Statistical Analysis

All statistics were performed using SPSS (version 16; SPSS Inc., Chicago, IL, USA). Results were expressed as mean ± SD, and statistical significance was set at *p* < 0.05. Two-tail unpaired* t*-test was performed to compare data from AT1 mRNA and protein expression between the two groups. Radioimmunoassay and real-time PCR data, except for AT1 mRNA expression, were analyzed by one-way analysis of variance (ANOVA) with a least square difference (LSD) multiple comparison test. The data obtained by ELISA and western blot analysis, except for AT1, were analyzed by ANOVA, followed by Bonferroni *post hoc* analysis.

## Results

### Surgical Stress Triggered Enhanced Hippocampal Formation of Ang II and Increased AT1 Activity

**Figure 1:**
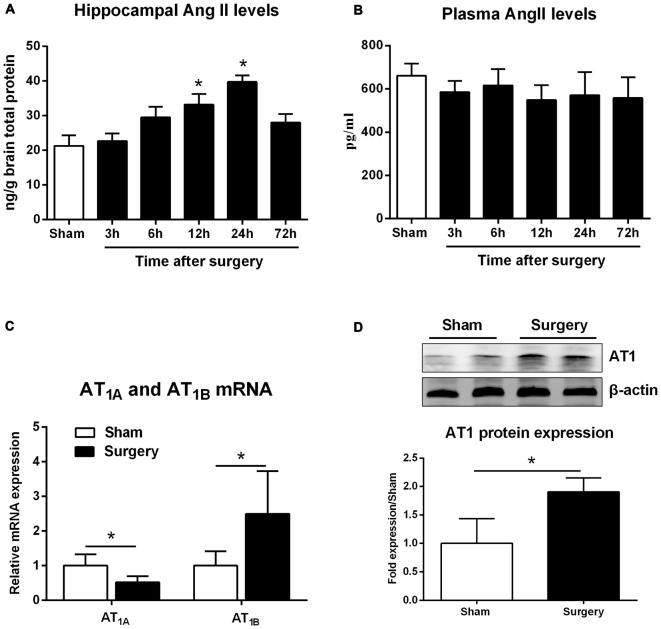
**Surgery effects on angiotensin II (Ang II) and Ang II receptor type 1 (AT1) levels in aged hippocampus.** Twenty-month-old rats received laparotomy under isoflurane anesthesia. **(A,B)** Kinetics of surgery-induced changes in Ang II levels in hippocampal tissues and blood plasma. Surgery enhances hippocampal formation of Ang II while Plasma Ang II levels were not altered following surgery. **(C)** Surgical stress enhances *AT_1A_* and attenuates *AT_1B_* mRNA abundance at 24 h after surgery. **(D)** Using a non-specific AT1 antibody, representative immunoblot (top) shows increased AT1 protein expression at 24 h after surgery when compared with sham rats. Values are mean ± SD (*n* = 6 vs. 4) for each condition. *p < 0.05, vs. sham.

Compared with sham animals, hippocampal Ang II expression increased at 12 h and reached a peak level at 24 h (Figure 1A; *p* < 0.05). Nevertheless, these changes occurred independently of circulating Ang II levels, which remained relatively stable after surgery (Figure 1B, *p* > 0.05). Surgery also caused downregulation of *AT_1A_* mRNA expression, whereas *AT_1B_* was significantly increased at 24 h post-surgery (Figure 1C; *p* < 0.05). Although rodents express two AT1 subtypes, most commercially available AT1 antibodies are non-specific (Benicky et al., 2012). Therefore, we chose sc-579 from Santa Cruz Biotechnology to detect total *AT_1A_* and *AT_1B_* expression and found that surgery significantly upregulated AT1 protein expression at 24 h postsurgery (Figure 1D; *p* < 0.05).

### Surgery Disturbed the Normal Balance between MMPs and TIMPs in the Aged Hippocampus

**Figure 2:**
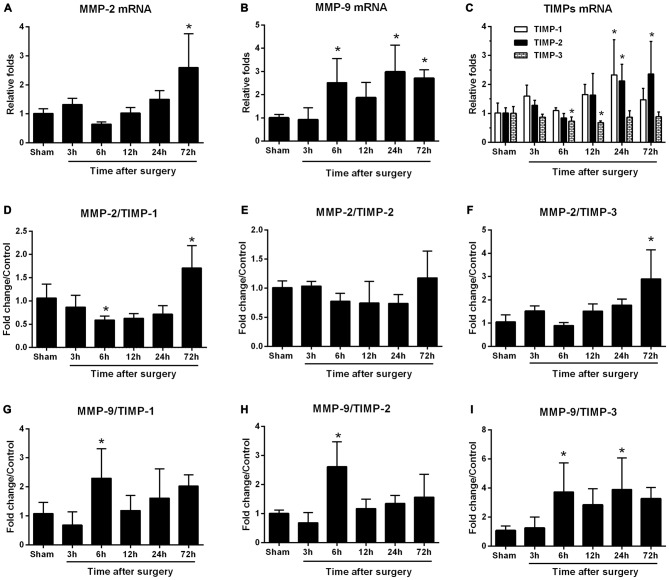
**Hippocampal matrix metalloproteinases (MMPs) and tissue inhibitors of MMPs (TIMPs) gene expression in response to surgery under isoflurane anesthesia.** Twenty-month-old rats received laparotomy under isoflurane anesthesia. The levels of MMPs, including MMP-2 and MMP-9, as well as three of their endogenous tissue inhibitors (TIMP-1, -2, -3), were dynamically determined at 3, 6, 12, 24, and 72 h after surgery using qRT-PCR. Kinetics of surgery- induced changes in transcript levels of MMP-2 **(A)**, MMP-9 **(B)**, TIMPs **(C)** and MMP:TIMP ratios **(D–I)** in the hippocampus are shown. Values are mean ± SD, *n* = 6. *p < 0.05, vs. sham.

We dynamically examined MMP-2 and MMP-9 mRNA expression at 3, 6, 12, 24, and 72 h following surgery. MMP-2 mRNA levels did not differ from sham levels until 72 h (*p* < 0.05; Figure 2A). MMP-9 mRNA levels significantly increased as early as 6 h and expression was sustained up to 72 h, except for the 12-h time point (*p* < 0.05 one-way ANOVA, with LSD *post hoc* test; Figure 2B). To evaluate MMP/TIMP balance, we simultaneously analyzed TIMP expression. Both relative TIMP-1 and TIMP-2 mRNA levels were increased at 24 h postsurgery, while TIMP-3 was significantly decreased within 6–12 h (*p* < 0.05 one-way ANOVA, with LSD *post hoc* test; Figure 2C). Then, the ratio of MMPs to TIMPs was calculated. We found that MMP-2/TIMP-1 was decreased at 6 h and then increased at 72 h (*p* < 0.05; Figure 2D), while MMP-2/TIMP-2 remained unchanged (*p* > 0.05; Figure 2E). However, MMP-2/TIMP-3 was stable in the early postoperative period (3–12 h, *p* > 0.05) and increased at 72 h (*p* < 0.05; Figure 2F). Compared with MMP-2/TIMPs, the MMP-9/TIMPs were much more disturbed, as evidenced by significant upregulation in MMP-9/TIMP-1, MMP-9/TIMP-2, and MMP-9/TIMP-3 as early as 6 h postsurgery (*p* < 0.05 one-way ANOVA, with LSD *post hoc* test; Figures 2G–I). Together, these results indicated that peripheral surgery induced a disrupted MMP/TIMP balance in the hippocampus of aged rats.

### The AT1 Antagonist Candesartan Suppressed Surgery-Induced Activation of Canonical NF-κB Signaling

**Figure 3:**
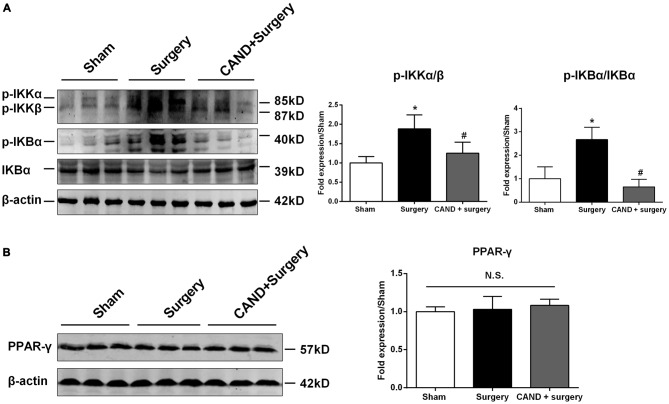
**AT1 antagonism inhibits surgery-induced activation of canonical nuclear factor-κB (NF-κB) signaling, but does not affect peroxisome proliferator-activated receptor-γ (PPARγ) expression.** Twenty-month-old rats received laparotomy under isoflurane anesthesia in the presence or absence of candesartan pretreatment. Prior to surgery challenge, candesartan was intraperitoneally administered at a dose of 0.1 mg/kg daily for 14 consecutive days. Results are expressed as fold change from the sham group. Representative western blots for p-IKKα/β, p-IκBα, IκBα, and PPARγ are shown on the left side of the bars. **(A)** Candesartan significantly blocks surgery-induced increases in the p-IKKα/β and p-IκBα/IκBα ratio at 6 h after surgery. **(B)** PPARγ expression was not altered under different experimental conditions. Values are given as means ± SD (*n* = 6) for each condition. *p < 0.05 vs. sham. ^#^*p* < 0.05 vs. surgery.

To explore the upstream signaling pathways leading to changes in gene expression of MMPs and TIMPs, we studied the involvement of the canonical NF-κB signaling and PPARγ expression at 6 h after surgery, a time point that showed a statistically significant disruption of MMP/TIMP balance at the mRNA level. Compared with the sham group, p-IKKα/β protein expression increased (1.9-fold; Figure 3A), which was attenuated by candesartan (*p* < 0.05 one-way ANOVA, with Bonferroni *post hoc* test; Figure 3A). Similarly, the ratio of p-IκBα to IκBα, an indicator of NF-κB activation, was much higher than in the sham group (2.7-fold; Figure 3A). This change was also significantly alleviated by candesartan pretreatment (*p* < 0.05 one-way ANOVA, with Bonferroni *post hoc* test). Because AT1 antagonism can confer anti-inflammatory action in the central nervous system (CNS) by activating other transcription factors, such as PPARγ (Garrido-Gil et al., 2012; Villapol et al., 2012), we examined whether PPARγ was involved in this process. We found no significant difference in PPARγ content in the hippocampus of rats under different experimental conditions (*p* > 0.05, one-way ANOVA; Figure 3B). Taken together, these data suggested that a disturbed MMP/TIMP equilibrium could be associated with NF-κB activation, but not PPARγ signaling.

### Candesartan Modulated Surgery-Induced MMP/TIMP Imbalance at the Translational Level

**Figure 4:**
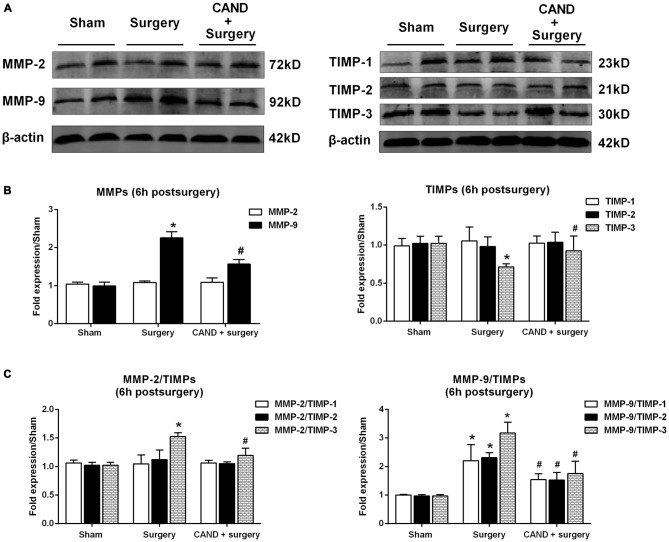
**AT1 antagonist candesartan pretreatment attenuates surgery-induced imbalance between MMPs and TIMPs at the protein level.** Twenty-month-old rats received laparotomy under isoflurane anesthesia in the presence or absence of candesartan pretreatment. Prior to surgery challenge, candesartan was intraperitoneally administered at a dose of 0.1 mg/kg daily for 14 consecutive days.** (A)** Representative bands of MMPs and TIMPs expression in the hippocampus at 6 h after surgery as detected by western blot analysis. **(B)** Semi-quantitative data showing protein expression levels of MMPs and TIMPs. Surgery upregulated MMP-9 protein expression and downregulated TIMP-3 expression.** (C)** The ratio of MMPs to TIMPs was calculated. The ratios of MMP-2/TIMP-3, MMP-9/TIMP-1, MMP-9/TIMP-2, and MMP-9/TIMP-3 were increased at 6 h post-surgery. These changes were all alleviated by candesartan treatment. Values are mean ± SD, *n* = 6. *p < 0.05 vs. sham. ^#^*p* < 0.05 vs. surgery.

Since there was a marked disturbance in MMP/TIMP equilibrium, accompanied by significant AngII elevation as early as 6 h after surgery, we further determined these changes at the translational level to evaluate the possible therapeutic effect of AT1 blockade. At 6 h after surgery, we found a significant increase in MMP-9 levels and a marked decrease in TIMP-3 content, as well as a consequent imbalance of MMP-2/TIMP-3, MMP-9/TIMP-1, MMP-9/TIMP-2, and MMP-9/TIMP-3 (*p* < 0.05 one-way ANOVA, with Bonferroni *post hoc* test; Figure 4). Importantly, these changes were attenuated by candesartan treatment (*p* < 0.05 one-way ANOVA, with Bonferroni *post hoc* test; Figure 4). These results reinforced that surgery-induced Ang II disrupted MMP/TIMP balance via AT1 receptors.

### Candesartan Improved Surgery-Induced Disruption of the BBB Integrity

**Figure 5:**
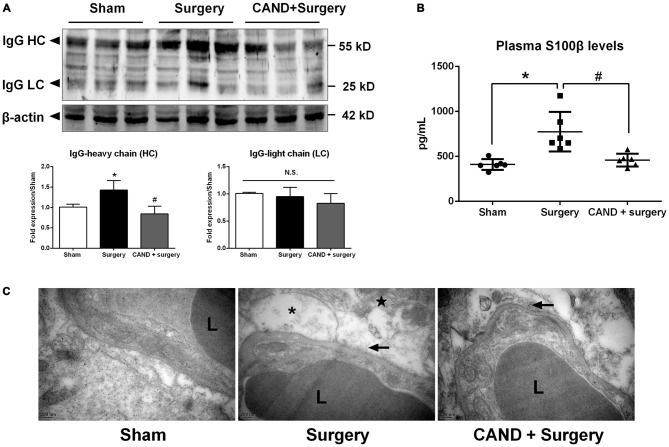
**Early inhibition of AT1 by candesartan improves the blood-brain barrier (BBB) permeability and attenuates the disruption of BBB ultrastructrure.** Twenty-month-old rats received laparotomy under isoflurane anesthesia in the presence or absence of candesartan pretreatment. BBB permeability was assessed by IgG expression in brain tissues and plasma levels of S100β. **(A)** Western blot analysis of IgG heavy chain (HC) and light chain (LC) expression indicated that candesartan reduced IgG-HC expression at 24 h after surgery (*n* = 6).** (B)** Serum S100β levels at 24 h after surgery. Enzyme-linked immunosorbent assay (ELISA) data revealed a significant increase in plasma S100β levels at 24 h post-surgery compared with sham, which was attenuated by candesartan pretreatment (*n* = 6). **(C)** Representative images of BBB ultrastructure from a coronal section through the hippocampal CA1 subfield (*n* = 4). Ultrastructure of the neurovascular unit was observed at 24 h after surgery using transmission electron microscopy. The capillary ultrastructure appeared nornal for the rats in the sham group. At 24 h postsurgery, the basal laminas partly collapsed (arrowhead), the perivascular spaces were enlarged. Swelling in astrocyte end-feet (*) and some swollen mitochodria (★) were also observed after surgery. Candesartan improves the above ultrastructure changes. L: lumen of blood vessel. Scale bar = 200 nm. *p < 0.05 vs. sham. ^#^*p* < 0.05 vs. surgery.

In the present study, we investigated functional and structural integrity of the BBB. BBB permeability was assessed by IgG expression in hippocampal tissue and S100β level in plasma. IgG, which is produced in the blood, is not present in the CNS when the BBB is intact. As a plasma-derived factor, IgG extravasation in the CNS may indicate disruption of the BBB (Ružek et al., 2011). Western blot studies in hippocampal lysates showed the presence of both heavy and LC IgG at the 24-h time point. There was no significant difference in LC expression. Nevertheless, expression of heavy chain (HC) IgG in the hippocampus, normalized to the sham, was significantly greater in the vehicle-treated group compared with the candesartan-treated group (*p* < 0.05 one-way ANOVA, with Bonferroni *post hoc* test; Figure 5A).

S100β is not specific to the brain. Nevertheless, at least 80–90% of the total S100β content is pooled within the brain, the remainder being located in several peripheral cells, including adipocytes, skeletal myoblasts and cardiomyocytes (Sen and Belli, 2007). It leaks into the blood stream when BBB integrity is compromised. In additon, higher levels of S100β are associated with reduced cognitive function in patients following different types of surgery (Linstedt et al., 2002; Leiendecker et al., 2010), brain injury and stroke (Herrmann et al., 2000). A one-way ANOVA of ELISA data revealed a marked increase in plasma S100β levels at 24 h after surgery (753.4 ± 238.9 pg/mL) when compared with sham (436.1 ± 49.3 pg/mL), which was attenuated by candesartan pretreatment (457.3 ± 79.7 pg/mL; *F*_(2,12)_ = 7.17, *p* < 0.001; Figure 5B). These results demonstrated that the surgical procedure induced higher BBB permeability, and AT1 antagonism reduced these changes.

To assess the structural integrity of the BBB, we used transmission electron microscopy. In the sham group, the ultrastructure of the basal laminas was continuous and integrated, the TJs and endothelial cells were normal (Figure 5C). Nevertheless, animals in the surgery group had impairments in the BBB at 24 h after surgery, consisting of local collapsed basal laminas, enlarged astrocytes end-feet, and dilated mitochondria and endoplasmic reticulum (Figure 5C). These changes were significantly attenuated by candesartan pretreatment, as evidenced by diminished angioedema surrounding the capillaries and obviously recovered basal laminas (Figure 5C).

**Figure 6:**
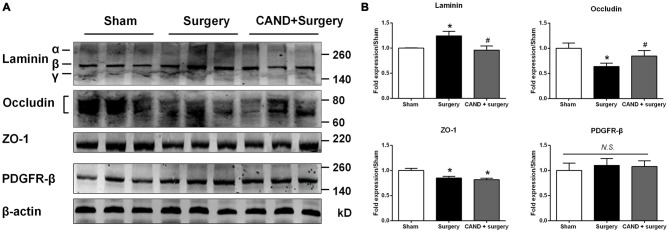
**AT1 antagonist candesartan pretreatment normalizes expression of BBB specific proteins after surgery. (A)** Representative western blots showing expression of basal lamina protein laminin, TJ proteins (occludin and zonula occludens-1 (ZO-1)), and the pericyte marker PDGFRβ in the hippocampus at 24 h after surgery in the presence or absence of candesartan pretreatment. **(B)** Densitometric analysis of western blots showing increased expression of laminin, reduced expression of TJ proteins occludin and ZO-1, and unchanged expression of PDGFRβ in the hippocampus of surgery rats compared with sham rats. Candesartan pretreatment significantly attenuates surgery-induced changes in expression of laminin and occludin, but not ZO-1. Values are mean ± SD, *n* = 6. *p < 0.05 vs. sham. ^#^*p* < 0.05 vs. surgery.

To further provide an anatomical basis for surgery-induced BBB disruption and the ability of candesartan to improve the BBB, we determined the protein expression of four BBB components. We found a significant increase in expression of laminin, a basement membrane marker, and a marked decrease in TJ proteins occludin and ZO-1, with no changes in PDGFRβ, a pericyte marker (*p* < 0.05 one-way ANOVA, with Bonferroni *post hoc* test; Figure 6). Candesartan pretreatment significantly preserved expression of occludin and suppressed laminin upregulation (*p* < 0.05; Figure 6), but did not ameliorate the decreased ZO-1 expression. Thus, the expression changes of occludin, ZO-1, and laminin may have contributed to impaired BBB integrity induced by disrupted MMP/TIMP balance.

## Discussion

Recent evidence has suggested an association between POCD following peripheral surgery and BBB disruption during this process (Terrando et al., 2011; He et al., 2012; Hu et al., 2014; Li et al., 2014; Zhang et al., 2014). The major focus of the present study was to determine hippocampal expressions of MMPs and TIMPs after surgery, and then to address whether a disturbed MMP/TIMP balance could serve as the underlying mechanism of surgery-induced BBB disruption. Here, we first demonstrate an imbalance between MMPs and TIMPs in line with increased hippocampal Ang II in aged rats after surgery. Moreover, the application of candesartan, an angiotensin-receptor blocker, restores the normal MMP/TIMP balance, thereby guaranteeing BBB integrity.

In the brain, Ang II is a multifunctional effector hormone that contributes to the regulation of cerebral blood flow and stress responses via AT1 receptor (Saavedra et al., 2011). While much is known about how an altered BBB contributes to neuroinflammation in POCD studies (Terrando et al., 2011; Zhang et al., 2014), no studies have addressed how surgery-induced Ang II expression might affect BBB function. Therefore, the effect of Ang II on BBB integrity remains a complex issue. Preclinical investigation from Kakinuma et al. (1998) reported impaired BBB function in angiotensinogen-deficient mice, suggesting astrocyte-dependent angiotensins are crucial for the functional maintenance of BBB. Similarly, clinical data also showed that astrocyte-derived Ang II stabilizes BBB permeability by threonine-phosphorylation of occludin in patients with multiple sclerosis (Wosik et al., 2007). In contrast, Kazama et al. (2004) reported that Ang II increases reactive oxygen species production in cerebral microvessels via NADPH oxidase. Later, Zhang et al. (2010) also reported Ang II-induced cerebral microvascular inflammation and increased BBB permeability via oxidative stress. Consistent with the latter two, we observed significant renin-angiotensin system hyperactivity in the hippocampus of aged rats after abdominal surgery, including increased Ang II content and upregulation of AT1. This was accompanied by increased BBB permeability, as evidenced by marked upregulation of IgG-HC expression in the hippocampus and increased serum S100β levels. More importantly, surgery-induced BBB dysfunction was ameliorated by chronic pretreatment with the AT1 antagonist candesartan, suggesting that Ang II modulates BBB permeability via activation of the AT1 receptor. Although the current study is not the first to suggest that Ang II plays a role in BBB modulation, the study is the first to directly link Ang II to changes in BBB integrity in POCD.

The main MMPs studied in relation to BBB disruption were MMP-2 and MMP-9, which can degrade the extracellular matrix of the basal membrane, as well as the TJ proteins. Furthermore, accumulating *in vitro* evidence has shown that in rat aortic smooth muscle cells (Guo et al., 2008, 2015), as well as in human umbilical vein endothelial cells (Jiménez et al., 2009), Ang II upregulates MMP-9 and MMP-2 expression. Therefore, we analyzed hippocampal expression of MMP-9 and MMP-2 and observed increased MMP-9, but not MMP-2, expression at 6 h after surgery. This was consistent with the findings by Browatzki et al. (2005) showing that Ang II induced expression of MMP-9, but not MMP-2, in human vascular smooth muscle cells obtained from human saphenous veins. A recent study reported that not only MMP-9, but also MMP-2, was increased in the aged hippocampus of another rat model of POCD (Hu et al., 2014). This discrepancy could be due to differences in sample time points (6 h vs. 24 h post-surgery), types of surgery (laparotomy vs. orthopedic surgery), and anesthetics (isoflurane vs. sevoflurane). In addition, we did not investigate the MMP activity in the hippocampus, although MMP activity seems to be associated with its level of protein expression (Zhang et al., 2012). MMP activity is tightly controlled, in both tissues and circulation, by endogenous inhibitors: TIMPs and α-2-macroglobulin (α2M; Baranger et al., 2014). Thus, further investigation of the enzyme activity would help to elucidate fully the complex biological effects of MMPs in POCD.

Generally, all TIMPs are capable of inhibiting all known MMPs; however, the efficacy of MMP inhibition varies dependent on the member of TIMPs, specific tissue, and local tissue environment (Baranger et al., 2014; Arpino et al., 2015). Although MMP-2/TIMP-2 and MMP-9/TIMP-1 ratios have been commonly used to evaluate the balance of MMPs/TIMPs in previous studies, the exact interaction between MMPs and TIMPs remains largely undetermined. Therefore we presented all the possible MMP/TIMP ratios in the current study, and found significant differences mainly exsit in MMP-2/TIMP-1 and MMP9 with all TIMPs at mRNA levels. Interestingly, the mRNA profile is equal to the protein content only for the MMP9 and TIMP3. Furthermore, we found TIMP-1 mRNA expression increased at 24 h after surgery to compensate for the earlier MMP-9 increase at 6 h. Nevertheless, it did not catch up with the increased MMP-9 protein expression. The inconsistency between transcription and translation levels maybe due to the variety of MMP activation mechanisms at post-tranlational level (Gu et al., 2002; Kar et al., 2010), which could affect the interaction between MMPs and TIMPs. Further investigation is required to elucidate the detailed interaction in the surgery challenged aged hippocampus.

More importantly, the MMP/TIMP imbalance at the translational level detected as early as 6 h after surgery corresponded with decreased expression of TJ proteins occludin and ZO-1 at 24 h post-surgery, as well as increased IgG extravasation into the brain parenchyma. We employed candesartan, an AT1 receptor blocker, which effectively normalized MMP-9 and TIMP-3 expression and restored their normal balance, thereby guaranteeing barrier integrity. These results, together with previous findings (Hu et al., 2014; Zhang et al., 2014), suggest that MMP/TIMP balance may be crucial for maintaining expression of TJ proteins. Interestingly, we observed an aberrant increase in basal laminin following surgery. Although the existing data revealed that cytokines, including tumor necrosis factor, transforming growth factor-β, and interleukin-1β, can upregulate laminins α4 and α5 in inflamed tissues (Sorokin, 2010), we do not exclude the possibility that leakage of serum protein IgG from loosened TJs between endothelial cells resulted in a compensatory upregulation of laminin in endothelial basement membranes.

Additionally, the acute disturbance of MMPs/TIMPs occurred at 6 h after surgery. This preceded the initial significant elevation of hippocampal Ang II expression at 12 h after surgery, although there was a non-significant increase in Ang II expression at 6 h post-surgery. However, this does not suggest that Ang II/AT1 signaling cannot be an activator of MMPs, because our results revealed that MMP/TIMP imbalance was normalized by AT1 blockade. The delayed kinetics in hippocampal Ang II content and earlier MMP/TIMP imbalance in response to surgical stress further suggest that other pathways that we previously reported, such as glucocorticoid receptor signaling (Tian et al., 2015), NADPH oxidase-dependent oxidative stress (Zhang et al., 2015), and NF-κB signaling (Zhang et al., 2014), may also participate in BBB damage following surgery.

We have also begun to investigate intracellular signaling mechanisms regulated by Ang II. NF-κB-mediated neuroinflammation plays critical roles in the POCD pathogenesis. Activation of the NF-κB pathway is associated with surgery-induced BBB and cognitive dysfunction (Terrando et al., 2011), and suppression of NF-κB has also been shown to attenuate surgery-induced BBB disruption, as indicated by IgG leakage (Zhang et al., 2014). In the present study, we found that prophylactic AT1 antagonism can significantly attenuate activation of the canonical NF-κB pathway and expression of the NF-κB downstream target gene MMP-9, suggesting that the Ang II/AT1/NF-κB pathway plays a crucial role in surgery-induced BBB dysfunction in aged hippocampus. Nevertheless, in view of angiotensinogen, the glycoprotein precursor of Ang II that is also a target gene of NF-κB (Brasier et al., 1990), we do not rule out the possibility that there was an interaction between Ang II/AT1 and NF-κB signaling. In fact, Ang II may activate multiple signaling pathways leading to activation of NF-κB and cAMP-response element binding protein (CREB), which further results in AT1 gene regulation (Haack et al., 2013). Further research on this issue may be warranted.

Accumulating evidence has revealed that AT1 blockers, including candesartan, activate PPARγ, which may be responsible for neuroprotective effects, independently of AT1 blocking actions (Erbe et al., 2006; Villapol et al., 2012). Interestingly, in the present study, we found that hippocampal PPARγ expression remained almost constant under different experimental conditions at 6 h after surgery. Our results suggested that PPARγ signaling did not appear to be involved in Ang II-induced MMP/TIMP imbalance in the aged hippocampus after surgery, and candesartan-induced BBB permeability was mediated by activity of AT1 and NF-κB blockade, but not PPARγ activation. Factors contributing to this discrepancy could include different stress types and concentration dependent effects.

**Figure 7:**
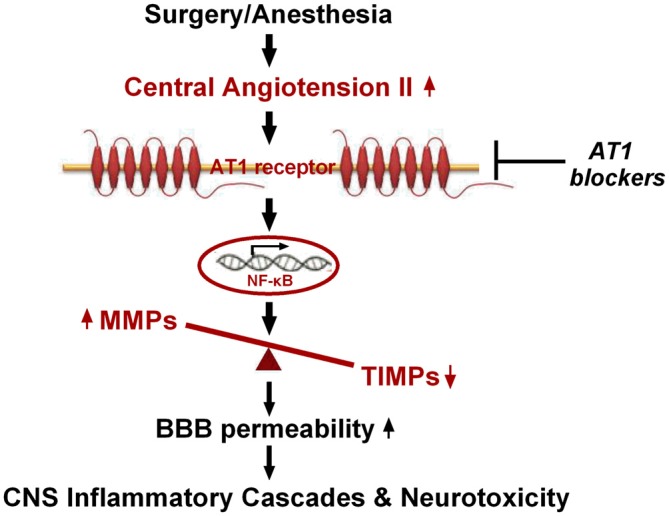
**Schematic illustration of the proposed mechanisms underlying BBB disruption in aged rats after surgery under isoflurane anesthesia.** Surgical stress increases expression of central Ang II and AT1. Ang II stimulation, dependent on AT1, leads to activation of NF-κB signaling and MMP/TIMP axis imbalance, further augmenting BBB permeability and neurotoxicity.

In conclusion, our findings present the first evidence, to our knowledge, that hippocampal disturbance of MMP/TIMP equilibrium contributes to surgery-induced BBB dysfunction in aged rats. This disturbance was associated with activation of NF-κB, but not PPARγ signaling, induced by Ang II elevation following surgical trauma (Figure 7). Thus, our findings further suggest that the Ang II/AT1/NF-κB pathway and regulation of the MMP/TIMP balance may lead to promising therapeutic targets for POCD.

## Author Contributions

Conceived and designed the experiments: ZL and NM. Performed the experiments: LL, YC, WW, HD, RX, LY, CN. Analyzed the data: YL. Drafted the original manuscript: ZL. Designed the experiments and edited the manuscript critically: DC, XG.

## Conflict of Interest Statement

The authors declare that the research was conducted in the absence of any commercial or financial relationships that could be construed as a potential conflict of interest.

## Abbreviations

POCD: postoperative cognitive dysfunction
MMP: matrix metalloproteinase
TIMP: tissue inhibitors of matrix metalloproteinase
Ang II: angiotensin II
AT1: angiotensin II receptor type 1
BBB: blood-brain barrier
AD: Alzheimer’s disease
CNS: central nervous system
NF-κB: nuclear factor-κB
PPARγ: peroxisome proliferator-activated receptor-γ.
